# Development and implementation of an interoperable local health information exchange platform for the attainment of universal health coverage in the Philippines

**DOI:** 10.3389/fdgth.2025.1650273

**Published:** 2026-01-30

**Authors:** Philip Christian Zuniga, Jan Jacob Carpio, Queen Anne Tayam, Patria Luwalhati Garcia, Andrew Lacsina, Jaztine Calderon, Jose Francisco Santiago, Dennis Batangan

**Affiliations:** 1 Computer Security Group, Department of Computer Science, University of the Philippines Diliman, Quezon City, Philippines; 2 Institute of Philippine Culture, Dr Rosita G Leong School of Social Sciences, Ateneo de Manila University, Quezon City, Philippines

**Keywords:** architecture, authoritative registry, digital health, interoperability, standards, universal health care

## Abstract

**Introduction:**

The implementation of Universal Health Coverage (UHC) in the Philippines requires the formation of Local Government Unit (LGU)-centric Health Care Provider Networks (HCPNs). A cornerstone of this reform is the development of secure, scalable, and interoperable digital health ecosystems. However, significant challenges persist, including fragmented and non-standardized health information systems, siloed data repositories, and limited interoperability among Electronic Medical Records (EMRs) and Hospital Information Systems (HIS) particularly at the LGU level. This paper presents the design and deployment of the Smarter and Integrated Local Health Information System (SMILHIS), a Local Health Information Exchange (LHIE) platform developed to address these fragmentation issues.

**Objective:**

This work aims to support LGUs in deploying an LHIE platform that improves health data exchange, enhances continuity of care, and strengthens local health systems. Specifically, the objectives are to: 1. Deploy a modular, open-source LHIE platform that enables real-time health data interoperability across primary, secondary, and tertiary facilities within LGUs; and, 2. Implement an HL7 FHIR-based integration model aligned with the OpenHIE architecture and national interoperability specifications.

**Findings and Conclusion:**

The implementation of SMILHIS in three pilot LGUs—Municipality of Pulilan in Bulacan, Cagayan de Oro City, and Pangasinan Province demonstrated both the technical feasibility and operational value of deploying an interoperable LHIE platform. Each pilot considered different use cases: in Pulilan, the integration of health data with regulatory and treasury systems required reconciling heterogeneous data structures; in Cagayan de Oro, the absence of authoritative registries within the HCPN created barriers to unified patient tracking; while in Pangasinan, the scale and geographic dispersion of the health network complicated real-time health service visibility. Despite these challenges, the SMILHIS platform integrated patient, provider, and facility registries, facilitated secure data exchange across multiple systems, and provided real-time referral and facility capacity dashboards. The key outcome of the study was the demonstrated ability of LGUs to implement an interoperable, standards-based LHIE that significantly reduced data duplication, improved referral coordination, and strengthened health system responsiveness. In addition to enhanced technological capability, the study also showed notable improvements in LGU digital health governance. Moreover, it contributed to strengthening human resource capacity through hands-on implementation experience and multi-sector collaboration, while promoting greater alignment with national government digital health standards. These institutional and technical gains highlight SMILHIS as both a practical solution and a capacity-building mechanism for sustainable digital transformation at the local level.

## Introduction

The enactment of Republic Act No. 11223 (1), or the UHC Act of 2019, marks a fundamental shift in the Philippine healthcare system, emphasizing the establishment of LGU-centric HCPNs. These networks position primary healthcare as the foundation of an integrated healthcare system, necessitating seamless exchange of health information among healthcare providers. Achieving this level of coordination requires the deployment of secure, scalable, and interoperable digital health infrastructures. Several EMRs are already available for LGUs to use. This include the DOH developed iClinicsys. Some EMRs were spinoffs from research projects like CHITS, while some are developed by private sector developers. However, despite this, it remains a challenge for LGUs to integrate these EMRs to form a functional LHIE that supports their respective HCPNs (2).

This article reports on the development and implementation of the Smarter and Integrated Local Health Information System (SMILHIS) (19), an interoperable, open-source Local Health Information Exchange (LHIE) platform piloted across three local government units (LGUs) in the Philippines. The paper documents how SMILHIS was designed and deployed, and more importantly, the operational lessons and governance insights that emerged during its implementation. By examining use cases in Pulilan, Bulacan; Cagayan de Oro City; and Pangasinan Province, the article highlights both the technical feasibility and institutional challenges of introducing a standards-based interoperability layer at the subnational level. Rather than serving as a project proposal or technical manual, this work contributes to the literature by providing practical experiences and lessons learned that can inform future efforts to strengthen health information exchange in resource-limited and decentralized health systems.

### Significance of the study

The significance of this work lies on the documentation of the first implementations of LHIEs in the Philippines to attain UHC. The deployment of a functional LHIE platform is expected to transform LGU healthcare service delivery by ensuring that health data remains accessible and interoperable across primary, secondary, and tertiary care settings. This initiative provides LGUs with a modular, vendor-agnostic digital health platform, allowing seamless integration with existing EMRs, HIS, and national health repositories such as the National Health Data Repository (NHDR) (3). Furthermore, establishing authoritative registries for patient identity, healthcare providers, and healthcare facilities will strengthen data integrity, streamline patient referrals, and enhance real-time monitoring of health services.

Ensuring the use of interoperable digital health systems ensures alignment with national UHC strategies, reinforces data privacy and security compliance, and empowers LGUs to transition from fragmented health IT systems to a unified, efficient digital health ecosystem. Additionally, an open-source implementation strategy will allow LGUs to reduce long-term costs, avoid vendor lock-in, and foster a collaborative digital health innovation ecosystem that supports local customization and continuous system enhancement.

This paper examines the challenges encountered by LGUs in achieving UHC, with a particular focus on the issues arising from data silos and the fragmentation of health information systems. The discussion will then shift to the SMILHIS technical architecture, emphasizing its role as an interoperability layer. Subsequently, the paper will present the findings from three pilot implementations of the SMILHIS platform, highlighting key insights related to system deployment, integration, and interoperability challenges. Finally, the concluding section will synthesize the lessons from these pilot studies, offering recommendations for scaling and sustaining an open-source, interoperable health information exchange framework within LGU-managed HCPNs.

### Health information exchanges

A Health Information Exchange (HIE) is a system that facilitates the secure, standardized sharing of health data across disparate health information systems. HIEs enhance care coordination, patient safety, and health system efficiency by facilitating the continuity of information across providers and care settings. Globally, HIEs are commonly implemented at the national level, typically overseen by a central authority and supported by unified infrastructure or centralized repositories. In contrast, federated HIEs preserve institutional data ownership, enabling semi-autonomous entities, such as hospitals, clinics, or local governments, to exchange data via a shared and interoperable layer.

Many countries have pursued national HIEs to bridge gaps in electronic health record (EHR) adoption and ensure interoperability across health systems. A 2019 global review of national HIE implementations across six countries found that, despite varying degrees of digital maturity, centralized health data exchange was considered essential for effective information sharing (4).

In the Philippines, early efforts to establish a national HIE began in 2015 with the Philippine Health Information Exchange (PHIE), but infrastructure limitations, governance challenges, and uneven digital readiness constrained progress. The passage of the UHC Act in 2019 provided renewed momentum, leading to the development of the NHDR, which was envisioned as the country's HIE. As part of the progressive implementation of a health information exchange (HIE) in the Philippines, the development of the NHDR, the National Health Data Dictionary (NHDD), and the Philippine Core Data for Interoperability (PHCDI) framework constitutes a foundational triad for enabling the secure and interoperable exchange of health information across the national health system. These three initiatives collectively serve as the structural backbone for achieving nationwide digital health interoperability. Each initiative is grounded in and directly responsive to the mandates of UHC law, which emphasizes the integration of health systems and the strategic use of ICT resources. The NHDR provides a centralized infrastructure for data aggregation and analytics; the NHDD standardizes data elements, definitions, and coding systems to ensure semantic consistency; and the PHCDI framework defines a harmonized set of core data elements for interoperable information exchange.

The NHDR is positioned as the central repository of health data in the country, aggregating and managing longitudinal and programmatic health records to support integrated service delivery, population health management, and national reporting. PhilHealth, as mandated by the UHC law, established the NHDR to support interoperability by ensuring that all health-related data in the country are submitted to the NHDR via interoperable HL7 FHIR APIs. Several submission models are being implemented in NHDR. One model is the centralized submission model, where all health facilities are required to submit data directly to the NHDR. Another implementation considers decentralized models, where health facilities can submit data through hubs.

Complementing the NHDR is NHDD. The NHDD serves as the authoritative and centralized catalog of all health data standards used within the Philippine health sector. As mandated by Department of Health Joint Administrative Order (DOH JAO) 2021-002, all health-related data collected, processed, and exchanged by government and private health information systems must conform to the standards and terminologies defined in the NHDD. This ensures consistency in the way health concepts are described and recorded, enabling both semantic and syntactic interoperability across diverse digital health systems. By standardizing data definitions, formats, and codes, the NHDD not only facilitates accurate data exchange and integration but also supports analytics, reporting, and decision-making at local and national levels. Ultimately, its alignment with the NHDR strengthens the broader digital health ecosystem and contributes to better health outcomes through more informed policy and clinical decisions. Further, the NHDD supports the attainment of semantic interoperability as it allow health data to be mapped to corresponding codes and terminologies hence developing a single understanding on the various data being used.

The PHCDI, on the other hand, plays a critical role in defining which specific datasets comprise key health objects necessary for health service delivery and data exchange. As part of its initial implementation, the PHCDI has focused on developing a standardized patient profile. This foundational dataset captures the essential information required to uniquely and consistently identify individuals within the health system. This initiative is significant in the context of standards localization. Instead of directly adopting global standards without modification, the PHCDI adapts and contextualizes them based on the actual needs, workflows, and constraints of local government units and healthcare providers in the Philippines. This ensures that the resulting standards are not only technically sound but also practical and applicable in real-world settings. By doing so, the PHCDI bridges the gap between international best practices and local realities, paving the way for more effective implementation of interoperable systems across varying levels of the health sector.

Subnational LHIEs, on the other hand, are less frequently implemented, mainly due to the high resource requirements and governance complexities at the local level. Notable exceptions include Ontario, Canada (5) and Xinjin County, China (6), where LHIEs were tailored to local contexts and successfully integrated diverse healthcare facilities. As per the review of existing digital health implementations in the Philippines, no similar technology is currently being utilized by LGUs.

Recent studies on HIEs have placed growing emphasis on implementation strategies for low-resource settings, offering lessons that could inform future SMILHIS deployments in geographically isolated and disadvantaged areas (GIDAs). Kiourtis et al. (7) and Vidakis et al. (8) investigate Bluetooth-based and other short-range wireless communication methods to facilitate secure health data exchange in situations where internet connectivity is intermittent or unreliable. This approach could strengthen SMILHIS's applicability in remote LGUs. Symvoulidis et al. (9) focus on resilient, low-latency data flows for emergencies, which could enhance SMILHIS's referral and coordination functions in disaster-prone or hard-to-reach areas. In parallel, Kiourtis et al. (10) and Kouremenou et al. (11) present structured HL7 FHIR-based data modeling processes to incorporate diverse health determinants into EHR systems. This method could improve the richness and interoperability of SMILHIS registries in contexts with fragmented data sources. Mavrogiorgou et al. (12) further demonstrate how Internet of Medical Things (IoMT) devices, combined with 5G network slicing, can generate and transform real-time health data into HL7 FHIR-compliant formats, opening opportunities for enhanced monitoring in rural health facilities.

While SMILHIS is currently positioned as a standards-based, LGU-managed HIE aligned with national frameworks (NHDR, NHDD, PHCDI), integrating these advances in low-bandwidth communication, IoMT data integration, and emergency-optimized exchange protocols could significantly expand its reach and resilience in future LGU implementations, especially in GIDA areas.

The SMILHIS platform introduces a novel, open-source approach to developing LHIEs, offering a digital health interoperability for LGUs. As a backend interoperability tool, SMILHIS enables data exchange across disparate health information systems through HL7 FHIR, ensuring compliance with national frameworks like the NHDR, NHDD, and PHCDI. Unlike one-off or siloed solutions, SMILHIS has been deployed in various locations and use cases, demonstrating its adaptability to diverse use cases while maintaining conformance with global and national specifications. This replicable framework bridges the gap between national digital health strategies and localized health service delivery, empowering LGUs to innovate while aligning with broader health system goals. SMILHIS is also not designed to be a vertical tool that supports individual programs; instead, it is designed to allow other systems to plug in and access other systems and registries.

Rather than functioning as a replacement for existing digital health tools such as EMRs, disease surveillance systems, or logistics platforms, SMILHIS operates as an integration backbone that enables health systems to interoperate. Its modular and open architecture allows LGUs to retain or adopt digital tools that suit their local needs while facilitating secure, standards-based communication between them. By serving as the integration layer, SMILHIS promotes data harmonization, reduces fragmentation and duplication, and supports more coordinated and efficient care delivery. This positioning makes SMILHIS a strategic enabler of a federated and scalable digital health ecosystem, supporting local autonomy, ensuring interoperability, and encouraging sustainable innovation through open-source collaboration.

In 2021, the Philippine Intellectual Property Office (IPOPHL) granted a utility model registration (1-2020-050024) to the eHatid Electronic Medical Record (EMR) system, formally recognizing its innovative design and functional utility. Building upon this registered technology, SMILHIS was developed as a derivative work of the EMR. While eHatid EMR primarily addressed the digital recording of patient encounters within health facilities, SMILHIS introduces a novel architecture and expanded interoperability framework, enabling seamless health information exchange across local government units, healthcare providers, and national registries.

## Current status of health data management at the LGU level

LGUs are the primary implementing bodies for healthcare service delivery at the grassroots level, ensuring that essential medical care is accessible to the general populace. Their responsibilities extend beyond service provision to include the maintenance and governance of health data, which is critical for operational efficiency and continuity of care. Given the dynamic nature of healthcare, LGUs must facilitate seamless access to patient information, especially within public health facilities under their jurisdiction. However, this obligation presents substantial challenges, particularly in environments where digital health systems are either underdeveloped or nonexistent. The absence of an EMR system within some facilities severely hampers clinical workflows, impeding the efficient documentation, retrieval, and exchange of health information. Without an EMR, patient data remains fragmented, often relying on paper-based records vulnerable to loss, degradation, and inefficiencies in storage and retrieval. Consequently, clinicians and healthcare personnel cannot leverage structured datasets that could enhance decision-making, epidemiological surveillance, and continuity of care (13).

The absence of an EMR is only one dimension of the challenge; the lack of interoperability among different health facilities exacerbates the issue further. Even in cases where individual LGU health centers have adopted digital systems, these solutions often operate in silos, preventing seamless data sharing between facilities within the same LGU or across different levels of care (2). Interoperability, defined as the ability of disparate systems to exchange, interpret, and use data coherently, is crucial for a coordinated healthcare system. Without adherence to established open standards, data exchange remains constrained by proprietary formats, inconsistent terminologies, and varied data governance policies. The inability to achieve interoperability results in redundant data entry, medical record errors, and patient care delays, particularly when individuals seek services at different healthcare centers or require referrals to higher-level facilities (14).

From a technical standpoint, implementing interoperable health information systems within LGUs necessitates a multi-tiered approach involving both policy and technology interventions. Standardizing health data structures, terminologies, and messaging ensures that digital health solutions can communicate effectively with one another. Additionally, adopting federated architectures, where data remains within local repositories but can be accessed through controlled mechanisms such as APIs and HIEs, can address concerns over data ownership. Furthermore, national systems, such as the NHDR, can serve as backbone infrastructures that facilitate data integration across the LGU health ecosystem.

## Technical components of the SMILHIS framework

**Figure 1:**
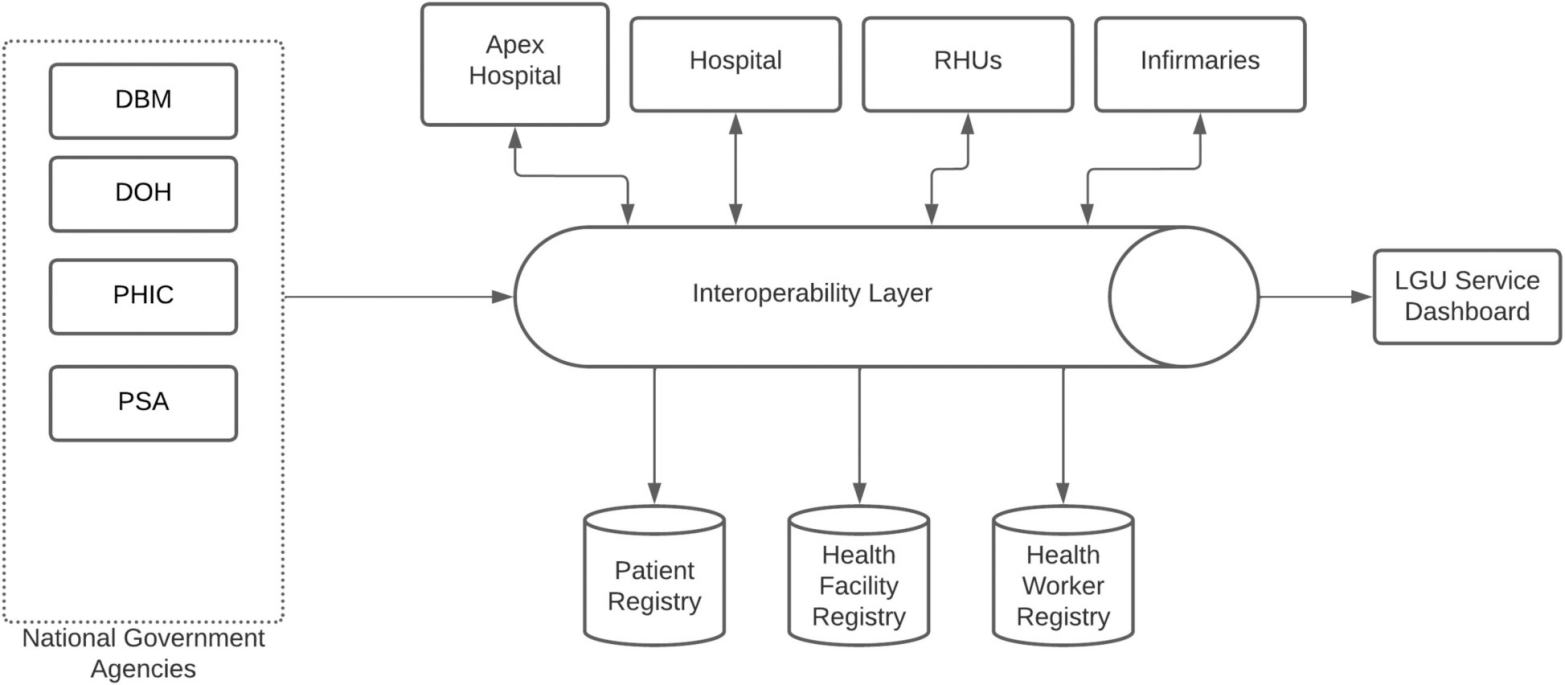
SMILHIS architecture (vanilla version).

The SMILHIS platform (Figure 1) is structured around three core components: architecture, authoritative registries, and standards, each of which is critical in ensuring a scalable and interoperable health information ecosystem for LGUs. The architecture is based on a modular, service-oriented framework that enables interoperability through APIs, ensuring seamless integration with existing digital health tools and systems. Consistent with the Philippine government's cloud-first policy, SMILHIS supports cloud-based deployments. The authoritative registries component serves as the backbone for data integrity and patient identity management, incorporating key national health databases such as the Client Registry (CR), Health Facility Registry (HFR), and Health Worker Registry (HWR), ensuring that all patient encounters, provider credentials, and facility records are harmonized across different systems. Lastly, adherence to standards ensures interoperability by implementing internationally recognized data standards, such as HL7 FHIR, and local frameworks, like the NHDD and PHCDI. These components enable real-time interoperability, fostering an integrated, patient-centric digital health ecosystem within LGUs.

### Architecture

**Figure 2:**
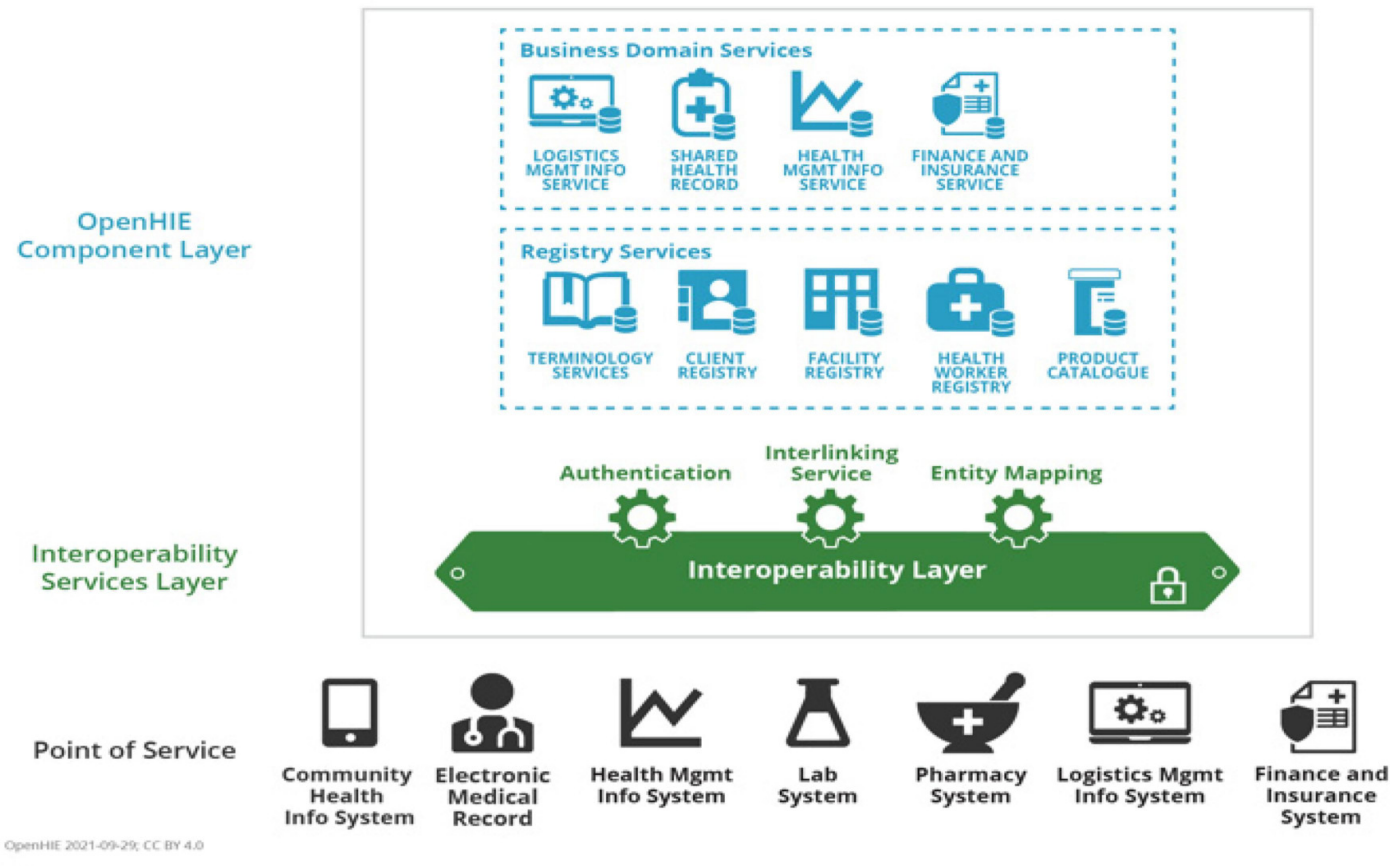
OpenHIE architecture. Reproduced from “OpenHIE Architecture Diagram” by OpenHIE Architecture Specification (https://guides.ohie.org/arch-spec), licensed under CC BY 4.0.

The platform is built upon the OpenHIE architecture (Figure 2), a globally recognized framework designed to support scalable HIE systems. OpenHIE provides a structured approach to integrating disparate digital health solutions by defining a modular and service-oriented architecture. This ensures that LGUs can adopt the platform without being constrained by a specific technology stack. By following OpenHIE principles (15), SMILHIS enables plug-and-play interoperability, allowing LGUs to integrate their existing health information systems—whether they be legacy EMRs, laboratory information systems (LIS), immunization registries, or referral systems—without requiring extensive modifications. This vendor-agnostic and open approach ensures that different LGUs with varying levels of digital maturity and infrastructure can still achieve real-time data exchange, thereby strengthening local HCPNs.

### Authoritative registries

SMILHIS has been developed to ensure full compliance with the OpenHIE architecture by incorporating multiple authoritative registries that function as single sources of truth for health data validation and management. These registries are fundamental in maintaining data integrity, minimizing redundancy, and facilitating interoperability across health information systems at the LGU level. By utilizing standardized and nationally designated data repositories, SMILHIS enhances data accuracy, consistency, and reliability, aligning with the principles of syntactic and semantic interoperability and structured health information management.

A key component of SMILHIS is the CR (Client Registry), which functions as a centralized identity management system for patient records. Previous implementations of LGU-managed registries, such as those developed for COVID-19 vaccination data, have provided temporary identity management solutions but have exhibited limitations in long-term sustainability, data consistency, and cross-LGU interoperability. To establish a more stable and unified identity verification system, SMILHIS proposes adopting the Philippine Identification System (PhilSys) as the primary national citizen registry. PhilSys provides a unique national identifier, which enables deduplication of patient records, facilitates streamlined identity validation, and ensures that individuals are consistently recognized across multiple healthcare facilities.

For health facility management, SMILHIS integrates the DOH National Health Facility Registry (NHFR), an authoritative database for facility classification and identification (16). This registry provides standardized facility identifiers, enabling consistent representation of hospitals, rural health units (RHUs), barangay health stations (BHS), private clinics, and specialty healthcare centers across digital health platforms. Integrating the DOH facility registry within SMILHIS ensures that healthcare service points are accurately indexed, service capabilities are correctly classified, and licensing data is consistently updated.

For the healthcare worker registry, SMILHIS incorporates multiple authoritative registries to ensure the validation of provider accreditation and licensing. The PhilHealth Provider Registry is a national reference database for PhilHealth-accredited healthcare professionals and facilities, ensuring that only registered providers participate in PhilHealth's health insurance programs. Additionally, the Professional Regulation Commission (PRC) is an authoritative source for verifying professional licensure, covering a wide range of healthcare practitioners, including physicians, nurses, and allied health workers. Integrating these registries within SMILHIS enhances provider authentication, facilitates digital workforce management, and ensures compliance with national licensing agencies' regulations.

Incorporating these authoritative registries within SMILHIS reinforces data governance, interoperability, and system-wide data integrity, ensuring that patient identity, facility information, and healthcare provider credentials are validated against nationally maintained reference datasets.

### Governance

The governance of SMILHIS was established through a multi-level, participatory framework that emphasized institutional ownership, policy alignment, and cross-sector coordination. A critical component in this structure was the formation of Technical Working Groups (TWGs) at the provincial, city, and municipal levels. These TWGs brought together key stakeholders from the LGUs, Municipal and Provincial Health Offices, ICT teams, planning departments, and healthcare providers. Their role was to guide the implementation process, validate workflows, define data governance protocols, and oversee technical deployment. Regular TWG meetings served as venues for harmonizing priorities, resolving interoperability issues, and ensuring that SMILHIS was responsive to both health and administrative needs.

In parallel, LGUs participating in the SMILHIS pilots enacted local ordinances and policies to institutionalize the use of the system and ensure legal compliance. These ordinances often mandated the adoption of SMILHIS as the official health information platform for the LGU, provided for budget allocation, and defined the roles and responsibilities of participating offices. Equally important was the drafting and signing of data sharing agreements (DSAs) between health facilities, laboratories, and LGU departments to enable lawful and secure data exchange. These agreements were aligned with existing legislation and established explicit provisions for data ownership, access rights, retention policies, and security protocols.

### Standards and terminology

The HL7 FHIR standard has emerged as a globally recognized framework for exchanging health data, representing structured data, and ensuring interoperability among diverse health information systems. As SMILHIS seeks to facilitate seamless integration across LGU health systems, the adoption of HL7 FHIR resources and APIs ensures structured, machine-readable health records that can be efficiently exchanged across healthcare facilities. HL7 FHIR's resource-oriented approach, which organizes clinical and administrative data into modular resources, allows LGUs to adopt flexible, scalable data architectures that align with national and international standards (17). Nationally, the NHDR serves as the anchor for HL7 FHIR implementation, as all health data submitted through it must use the HL7 FHIR specification.

In that sense, SMILHIS plays a critical role in the deployment of the NHDR by serving as a trusted source of validated, structured data at the LGU level. SMILHIS can submit clean, formatted data (e.g., on encounters, referrals, immunizations, and service delivery) directly to the NHDR via APIs. In return, the NHDR enhances the value of SMILHIS by enabling feedback loops and comparative analytics, allowing LGUs to benchmark their performance against regional or national indicators. This bidirectional exchange fosters a culture of data utilization, enhances alignment between local and national priorities, and ultimately contributes to a more responsive, integrated, and data-driven healthcare system.

Unlike the full OpenHIE stack, the current SMILHIS implementation does not include a dedicated, localized terminology service. This design choice was intentional. Terminology services are highly specialized components that require sustained national-level governance, continuous content curation, and rigorous alignment with evolving health standards. Implementing such a service at the LGU level would risk divergence from national specifications, create unnecessary duplication of effort and technical overhead on the side of LGUs. Instead, SMILHIS is designed to integrate seamlessly with the terminology service that will be adopted and maintained by the National Government. By deferring to a centrally managed national service, SMILHIS ensures that all terminology bindings, code validations, and mappings remain consistent across the country, thereby promoting long-term semantic interoperability without introducing parallel or conflicting terminologies at the local level.

## Design and development methodology

This section presents both the overarching development methodology of SMILHIS and the standardized implementation approach employed across pilot sites. Despite the diversity of use cases, a consistent implementation framework was applied, demonstrating that core components of SMILHIS can be reused and contextually localized. This highlights the system's modular architecture and its capacity to support scalable and adaptable deployment across varying local government settings.

In the Philippines, foundational efforts to establish a Health Information Exchange (HIE) began with the prototype implementation of the PHIE in 2015 (18). This initiative introduced a national HIE architecture based on the OpenHIE framework, utilizing an enterprise service bus as the central integration layer. The PHIE prototype served as a key reference model, informing the architectural design of SMILHIS. During the initial pilot in Pulilan, Bulacan, this architecture was adapted and localized, effectively replicating the PHIE prototype at the local government level. The replication involved three core components: (1) implementing shared registries for patients, health workers, and health facilities; (2) establishing an interoperability layer to facilitate standards-based data exchange; and (3) developing edge applications to provide user-facing interfaces for interacting with the system.

### Authoritative registries

The development of authoritative registries within SMILHIS centers on establishing standards-based databases for managing core entities: patients, health workers, and health facilities. In the early stages of implementation, the data fields in these registries were intentionally limited to those required by the specific use cases of pilot LGUs. This pragmatic approach enabled rapid deployment and allowed for context-specific customization.

As national digital health initiatives advanced—particularly with the formal adoption of HL7 FHIR as the national standard for health information exchange and the release of the PHCDI—the need to align with standardized data models became increasingly critical to ensure both semantic and structural interoperability. To address this, subsequent SMILHIS implementations adopted HL7 FHIR resources as the canonical data model for registry development: the **Patient** resource for the client registry, the **Practitioner** and **PractitionerRole** resources for the health worker registry, and the **Organization** resource for the health facility registry, consistent with the National Health Facility Registry (NHFR).

Standardizing the fields within these registries to conform to nationally accepted specifications was a key task in the pilots. In Pulilan, the patient registry and laboratory data fields adhered to the structure defined in the PHIELite implementation (DOH–PHIC JAO 2016-0003), an earlier initiative by the DOH and PhilHealth to standardize patient-related data elements. These fields were then mapped to corresponding HL7 FHIR resources. In later deployments, such as in Cagayan de Oro and Pangasinan, the registries were designed directly around data elements defined in the NHDD, with subsequent mapping to FHIR resources. Where NHDD already contained dictionary entries—such as standard identifiers for common laboratory tests, facility classifications, and provider roles—direct mapping was performed. For concepts not yet present in NHDD, temporary local codes were used and flagged for future alignment once official entries are published, ensuring that registry content remains interoperable and future-proof.

Over time, these registries have not only been made interoperable through the use of FHIR but have also been increasingly aligned with national profiles and terminologies. Notably, the client registry is currently being redeveloped to conform to the PHCDI Patient Core Profile, ensuring complete alignment with national interoperability standards and enabling seamless integration with other national and local health information systems. This progression marks SMILHIS's transition from a pilot-driven initiative to a standards-aligned, scalable architecture for health information exchange.

### Interoperability layer

As outlined in earlier sections, the initial implementation of SMILHIS closely mirrored the architecture of the Philippine Health Information Exchange (PHIE), which employed an enterprise service bus (ESB) as its interoperability layer. An ESB functions as a middleware solution that facilitates communication between disparate systems by handling tasks such as message transformation, routing, protocol mediation, and complex service orchestration. This architecture was effective in a national context where heterogeneous systems required advanced integration patterns. However, in the context of the Pulilan pilot—a localized implementation at the LGU level—the complexity and overhead introduced by the ESB were found to be unnecessary and burdensome. The LGU's business processes did not require advanced orchestration features, making the ESB both technically and operationally excessive.

To address this, the interoperability layer was re-engineered using an HL7 FHIR server, specifically the open-source HAPI FHIR server. This shift was informed by consultation and validation with the local IT team. The HAPI FHIR server provided a lightweight, standards-compliant platform capable of storing, retrieving, and validating HL7 FHIR resources with RESTful APIs. Unlike an ESB, which requires configuration of multiple integration components and adapters, the FHIR server offers a more streamlined architecture well-suited for the relatively straightforward workflows at the LGU level. Additionally, HAPI FHIR's open-source nature aligns with the overall design philosophy of SMILHIS, promoting vendor neutrality, cost-effectiveness, and community-driven development. This architectural simplification not only reduced deployment and maintenance burdens but also accelerated the implementation timeline, allowing LGUs to quickly establish interoperable health information systems that conform to national data standards.

### Edge systems

The edge systems in the SMILHIS architecture were designed as modular user interface layers that interact directly with end-users, facilitating access to the underlying interoperability infrastructure. These systems are context-dependent and vary based on the specific deployment scenario and local use case. A lightweight, web-based navigation and landing module was developed as a common entry point for LGUs, providing authenticated access to various system components and registry services. This front-end shell is intentionally minimalistic to reduce resource consumption while enabling modular expansion based on LGU-specific workflows.

In the Pulilan pilot, the edge system included a customized extension of the eHatid EMR system, which incorporated a file upload mechanism to enable private laboratories to submit diagnostic results in a structured format. Additionally, internal web modules were developed and deployed for the Municipal Health Office (MHO) and City Treasury Office, enabling secure, role-based access to relevant workflows such as health certificate validation and permit processing. For the Cagayan de Oro and Pangasinan pilots, edge systems included real-time data visualization dashboards that aggregated HL7 FHIR resource data from shared registries and facility-level systems. These dashboards provided operational intelligence on referral trends, facility utilization, and service availability, supporting both administrative decision-making and public service navigation.

### Pilot deployment of SMILHIS

Pilot implementations of SMILHIS follow a structured, step-by-step process (Table 1) that integrates design thinking, technical development, and capacity building to support the successful deployment of an LHIE at the LGU level. The approach begins with a strong emphasis on user-centered design, followed by iterative system building and continuous stakeholder engagement. The following are the usual deployment and development methodologies of SMILHIS. Constant validation with stakeholders has also been factored into the methodology, and hence it is possible that such an implementation methodology will still evolve in future implementations.

**Table 1:** Implementation steps.

Steps	Description
0) Design Thinking Workshop	SMILHIS pilot implementations has started with the conduct of a design thinking workshop for the developers to identify the most important use case where SMILHIS can be modified. Given that SMILHIS was designed to be use case agnostic, the workshop has allowed us to customize and localize SMILHIS based from priority use cases hence providing more value to stakeholders
1) Project Engagement and Mobilization	Following the design workshop, formal project mobilization takes place. This involves securing stakeholder commitments, forming TWGs, and aligning the project with local and national health governance structures. It also includes the identification of implementation sites, partners, and local champions to ensure institutional support throughout the deployment. Technical and functional requirements are initialized in this stage.
2) Joint Architectural Design session	Based on the analysis, the most suitable use case are identified. These are validated during Joint Application Design (JAD) sessions, which serve to confirm technical and functional requirements with local stakeholders. JAD has allowed the team to further refine the system.
3) Turnover	After having a clear use case, the system will be turned over to IT developers of the LGU, with minimal work done by the SMILHIS developers. This is done to promote system ownership of the LGU
4) Customization	This step is done both by the LGU's IT team and the SMILHIS developers. Customization is done to support the priority use case identified during the earlier phase of the deployment.

## Results of pilot implementation

Three pilot implementations (Table 2) of the SMILHIS project have been successfully executed. Each leverages the SMILHIS framework while addressing varied use cases. The pilot areas were specifically selected due to their varying capabilities as LGUs. Pulilan, Bulacan, is a small municipality, while Cagayan De Oro (CDO) is a highly urbanized city, and the Province of Pangasinan is a province. Despite the differentiation in use-case applications and LGU capabilities, the underlying platform remained consistent across all deployment sites to ensure interoperability, scalability, and adherence to standardized health information exchange protocols. The strategic selection of pilot sites allowed for comparative assessments of system adaptability, data integration efficiency, and contextual deployment challenges within different local government unit (LGU) environments.

**Table 2:** Summary of pilot implementations.

Pilot Site	Specific Use Case	Data Sources	Data Elements Processed
Pulilan, Bulacan	End-to-end digitalization of sanitary permit applications.	Municipal Health Office (MHO) City Treasury Office—Private laboratories	Applicant demographic data Health certificate issuance Laboratory test results Community Tax Certificate (Sedula) info Payment transactions
Cagayan de Oro City	Integrated patient referral and continuity of care system	Primary care centers LGU-operated hospitals Apex referral hospitals Shared registries	Patient demographic and clinical data Referral records Provider and facility IDs Patient encounter history UHC enrollment validation
Pangasinan Province	Deployment of a real-time health service dashboard	RHUs, infirmaries, provincial and district hospitals Provincial Health Office—EMRs and HIS	Real-time facility capacity Available services per facility Validated provider/facility info Referral volume and traffic data—Service gaps

### Pilot 1: Pulilan, Bulacan

**Figure 3:**
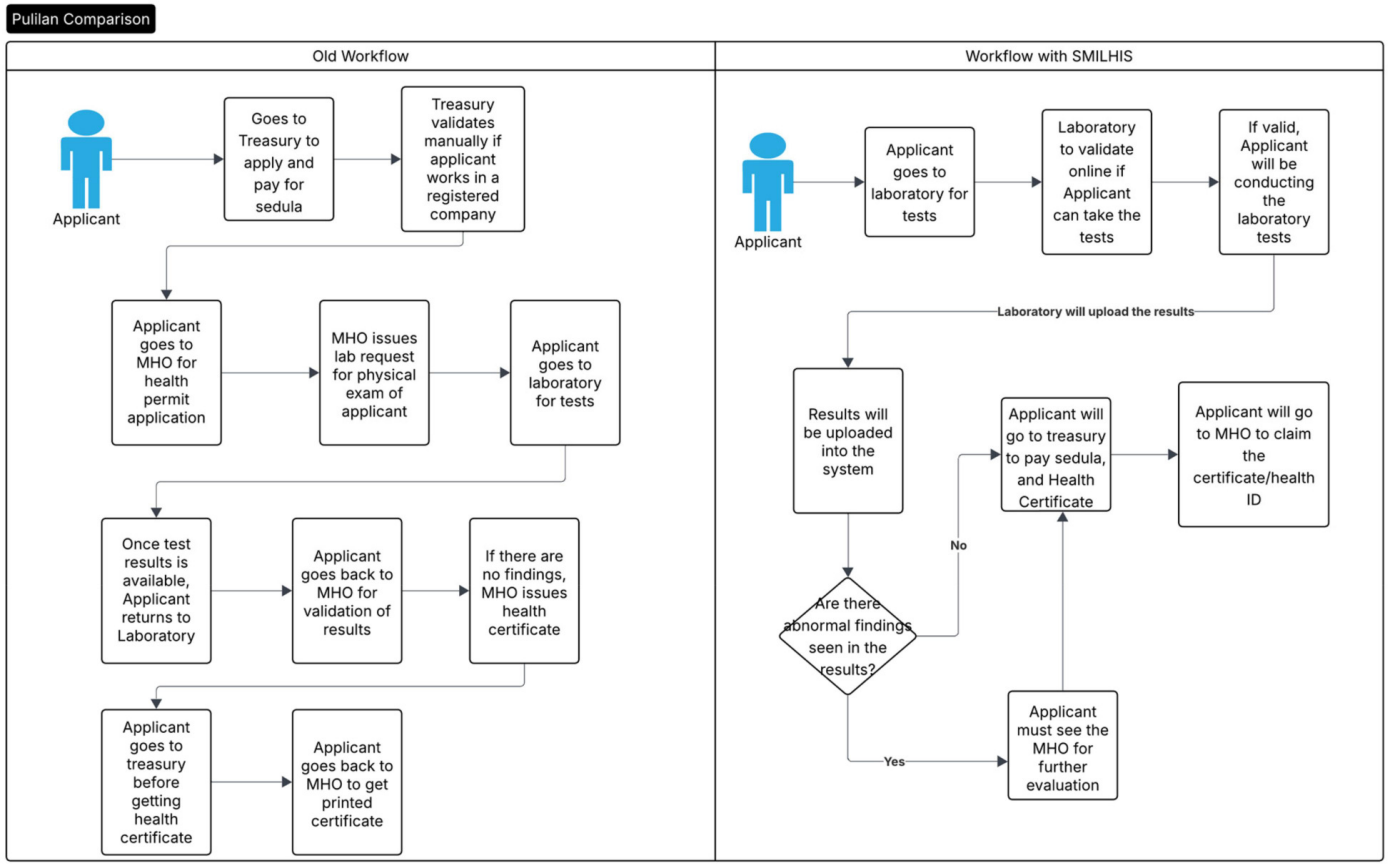
Comparison of the old manual process with the SMILHIS integrated process.

The pilot implementation of SMILHIS in Pulilan, Bulacan, focused on optimizing citizen-to-government interactions, particularly in managing and utilizing health data for regulatory processes. The selected use case demonstrated the end-to-end digitalization of the sanitary permit application, a crucial requirement for businesses and employment that necessitates coordination across multiple government offices. Traditionally, the process involves several manual steps, including payments at the City Treasury Office, acquisition of a Community Tax Certificate (Cedula), issuance of a health certificate by the MHO, and laboratory testing conducted by an accredited private laboratory. The pilot aimed to streamline these procedures by leveraging a health information interoperability framework, facilitating seamless data exchange between stakeholders. The chart in Figure 3 compares the previously manual process with the process once SMILHIS is adopted.

A JAD was conducted, which involved the MHO, IT Office, Treasury Office, and Planning Office. During this JAD, it was identified that integrating several office workflows is necessary to ensure better system implementation and to automate the initially manual process of applying for a sanitary permit.

An HL7 FHIR server became the interoperability layer, utilizing health data standards to ensure compatibility between health records, treasury transactions, and laboratory results. The laboratory engaged for the pilot did not use a digital laboratory information system; therefore, an ETL system was deployed to enable the laboratory to upload its laboratory results using a template developed by the SMILHIS team.

Integrating private laboratory systems into the municipal health infrastructure introduced a paradigm shift in the secure handling of health data. The risk of document forgery or tampering was mitigated by eliminating the need for physical submission of test results and reducing human intervention in the data processing process. A secure transmission protocol was established to enable direct electronic submission of laboratory test results to the MHO, ensuring data integrity and authenticity.

The pilot integrated data sources from the MHO, private diagnostic laboratories, and treasury systems, each contributing distinct but interrelated datasets to the permit issuance process. Key data elements processed included applicant demographic information, health certificate issuance records, laboratory test results (e.g., drug testing, chest x-ray, CBC), Cedula and tax payment details, and digital proof of payment.

The implementation utilized a range of HL7 FHIR resources to facilitate structured and interoperable data exchange throughout the sanitary permit application process. The **Patient** resource captured applicant demographic details, serving as the core reference for all linked health and administrative records. The **Encounter** resource documents interactions between applicants and municipal health personnel, particularly during the issuance of health certificates. **Observation** resources were used to represent laboratory test results, including drug tests, chest x-rays, and complete blood counts, which were uploaded via an ETL system by the partner private laboratory. The **Practitioner** and **Organization** resources represented municipal health officers and the various facilities involved, including the MHO and private labs. To facilitate the digital issuance of health certificates, the **DocumentReference** resource was used to manage and share signed digital documents, replacing manual submissions.

Additionally, **Questionnaire** and **QuestionnaireResponse** resources were utilized to capture structured application data, including health declarations and administrative inputs. While treasury and tax payment systems were external to SMILHIS, relevant transaction metadata were referenced using custom extensions within FHIR resources to maintain interoperability. Together, these resources enabled a seamless and secure data flow, reducing redundancy, enhancing data integrity, and significantly streamlining the citizen-facing permit process.

### Pilot 2: Cagayan de Oro city

The pilot implementation of SMILHIS in Cagayan de Oro focused on establishing a digital health architecture that enables data exchange between primary care centers, LGU-operated hospitals, and the apex referral hospital. As one of the UHC pilot sites in the Philippines, the city has been a pilot in developing integrated HCPNs, requiring an interoperability framework to facilitate referrals and continuity of care. Given the multiple tiers of healthcare facilities, ensuring a structured and interoperable referral system was essential for optimizing service delivery and enhancing patient outcomes. The SMILHIS implementation aimed to create a health information exchange (HIE) architecture that enables the secure sharing of patient data across care settings, while ensuring compliance with national eHealth policies.

During the JAD workshops with the CHO, IT Office, Civil Registry office, City Hospital management, and local insurance office, it became evident that the existing health service delivery network in the city was highly complex, primarily due to the presence of disparate EMRs and HIS operating across different levels of care. One key gap identified during the JAD is the siloed point-to-point integration protocol currently in place. As such, a key contribution of SMILHIS was the implementation of an OpenHIE-based architecture, which provided a framework for integrating heterogeneous health information systems using FHIR. This allowed for the establishment of shared registries, ensuring that patient records could be consistently linked across primary, secondary, and tertiary healthcare facilities.

Another innovation introduced by SMILHIS was the development of a Registry dashboard, which provided real-time visibility into the distribution of healthcare resources across the LGU. By aggregating data from various facilities, the dashboard enabled policymakers and health administrators to identify gaps in service coverage, optimize resource allocation, and improve facility-level decision-making. Additionally, integrating patient, facility, and health worker registries enabled more efficient citizen profiling and validation, a necessary component of UHC implementation. Through these registries, healthcare providers can access accurate and up-to-date patient demographic and clinical information, reducing record duplication and enhancing care coordination across the HCPN.

**Figure 4:**
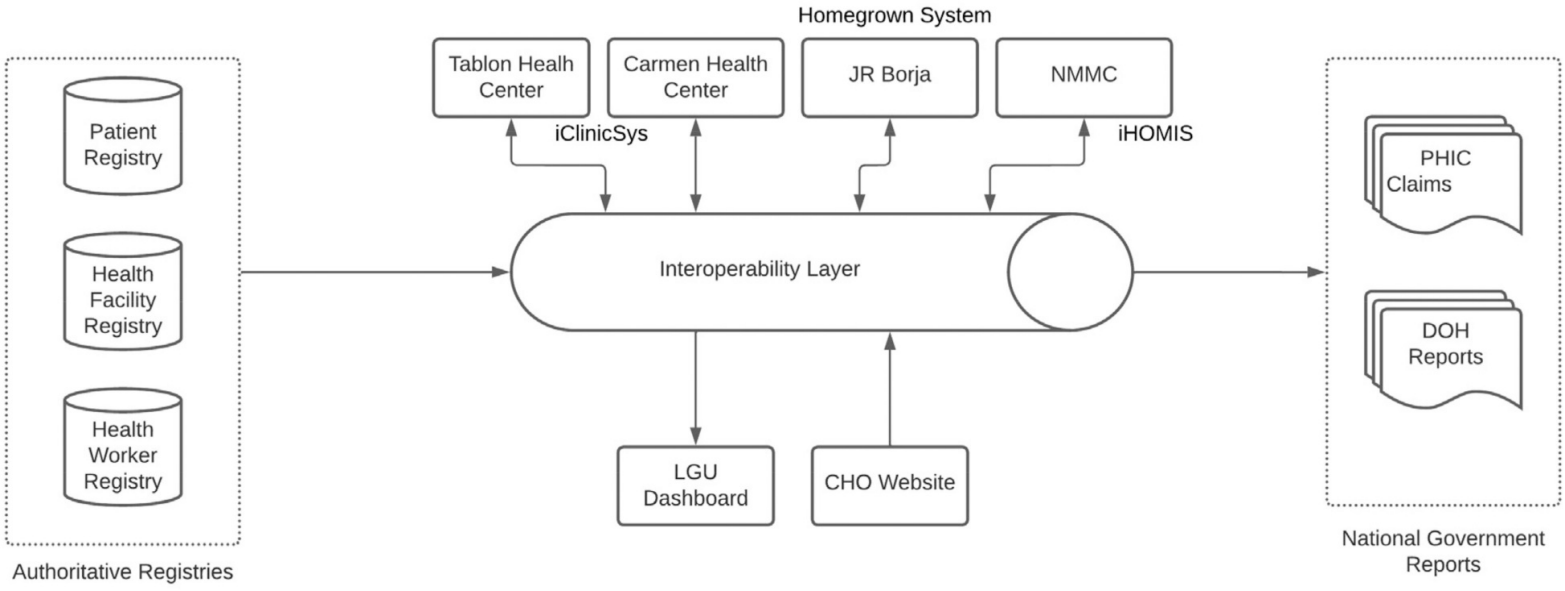
CDO architecture.

To support the referral use case, SMILHIS drew from multiple data sources, including shared registries (e.g., Patient, Provider, and Facility Registries), as well as administrative databases managed by the City Health Office and DOH partners. The patient registry originated from existing COVID-19 vaccination records maintained by the City. The facility registry came from the NHFR, while SMILHIS developed a separate registration sheet for the health worker registry. These registries became accessible to various health facilities, ensuring that the referral use case could be implemented. Figure 4 shows the refined architecture, illustrating how the registries became accessible to various health facilities in real-time.

Key HL7 FHIR resources utilized in this pilot included the **Patient** resource, which served as the basis for the patient registry by consolidating demographic data initially sourced from existing COVID-19 vaccination records. The **Practitioner** and **PractitionerRole** resources enabled the creation of a health worker registry, supporting accurate identification and role assignment of healthcare providers across facilities. The **Organization** resource was used to build a facility registry aligned with the National Health Facility Registry (NHFR), ensuring that all health facilities within the network could be uniquely identified and referenced. These three registries—patient, provider, and facility—were integrated into a real-time, shared infrastructure accessible across the local health system, significantly enhancing citizen profiling, record validation, and cross-facility coordination. The Encounter resource was also incorporated to track service events across care settings, linking patients, providers, and facilities in a structured and interoperable way. This registry-driven architecture laid the foundation for more efficient referral processes and continuity of care within Cagayan de Oro's HCPN.

### Pilot 3: province of Pangasinan

The SMILHIS pilot implementation in Pangasinan focused on enhancing the visibility of health services by developing a real-time health referral dashboard. Given that Pangasinan is one of the largest provinces in the Philippines, both in terms of land area and population, with a vast and centralized health service delivery network composed of RHUs, infirmaries, and government hospitals, ensuring proper service allocation was a significant challenge. The province required a mechanism to provide citizens and healthcare administrators with timely and accurate information on available healthcare services and resources across its extensive network. By leveraging the SMILHIS framework, the pilot aimed to establish an integrated data-sharing ecosystem that could inform both the public health system and the LGU about the distribution of healthcare services within the province.

**Figure 5:**
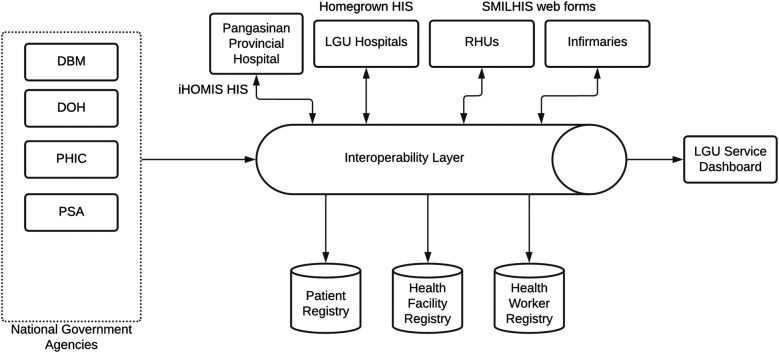
Pangasinan architecture.

**Figure 6:**
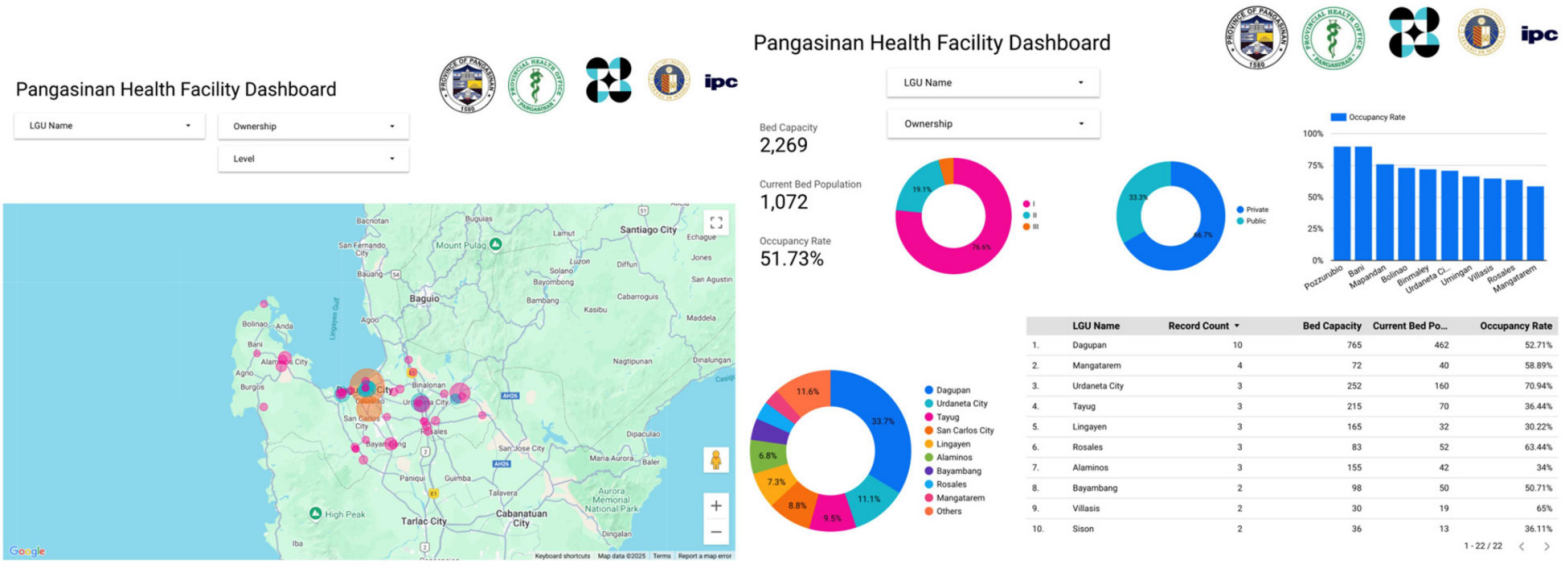
Sample dashboard.

The necessity of this dashboard became evident during the design thinking workshop and JAD conducted with the PHO, where stakeholders (PHO, Pangasinan Provincial Hospital, Civil Registry, and Social Work) emphasized the need for real-time health facility data to improve patient service navigation and resource allocation. Citizens often face difficulties identifying which health facilities could cater to their needs, leading to delays in accessing essential healthcare services. Similarly, provincial health administrators struggled with real-time monitoring of facility capacity, service availability, and supply chain gaps. Through SMILHIS, a health referral dashboard was developed as a centralized platform that aggregated and visualized data from multiple EMRs and HIS, ensuring that healthcare providers and the public had access to reliable, real-time facility information. In Figure 5, we can see how the OpenHIE architecture was localized in Pangasinan, with the different local registries showing, and how the data from registries and facilities can be transmitted to the sample dashboard (Figure 6).

One of the primary benefits derived from implementing the SMILHIS-powered dashboard was improved patient decision-making. By making health facility availability data accessible to the public, citizens could determine where to seek care based on service availability, reducing congestion in overburdened facilities and optimizing the use of underutilized health centers. The system also ensured that the data flowing into the dashboard was validated through authoritative registries, maintaining high data accuracy and preventing misinformation. The ability to visualize referral patterns also allowed the provincial government to identify gaps in healthcare service distribution and strategically plan interventions to enhance service accessibility.

To realize this use case, the system consolidated data from multiple sources, including facility-level inputs, Provincial Health Office (PHO) records, and existing HIS platforms used across RHUs and hospitals. The key data elements processed included real-time facility capacity (e.g., bed occupancy, service availability) as well as validated provider and facility information from shared registries. For facilities without an HIS—primarily rural health units and local infirmaries—a lightweight data entry module/web form was developed within SMILHIS to capture essential information on capacity and resources. Larger hospitals with established HIS platforms were integrated directly via available APIs, enabling automated data exchange. The dashboard aggregated all inputs using standardized formats and FHIR-compatible APIs, ensuring timely updates and reducing reliance on purely manual reporting. By integrating authoritative registries, the system maintained high levels of data accuracy and consistency, thereby mitigating the risks of misinformation and enabling strategic resource planning.

The SMILHIS pilot in Pangasinan leveraged key HL7 FHIR resources to power the real-time health referral dashboard, improving service visibility and optimizing resource allocation across the province's extensive health network. The **Organization** resource formed the foundation of the facility registry, capturing validated data on RHUs, infirmaries, and hospitals. The **Practitioner** and **PractitionerRole** resources supported the health worker registry, ensuring accurate identification and role mapping of providers. **Encounter** resources were utilized to track referral events and patient interactions, allowing for the analysis of service utilization and referral patterns. Facility capacity and service availability data—collected either through the SMILHIS input module or via API integration—were mapped to the **Location** and **HealthcareService** resources for standardized reporting. These structured inputs were then aggregated and visualized in the dashboard, providing both public users and provincial administrators with timely, validated data on the health system. By leveraging standardized resources and shared registries, the system established a reliable foundation for informed decision-making and enhanced patient navigation throughout Pangasinan's healthcare network.

## Results and findings

This section presents the results and discussion from the initial implementation of the SMILHIS platform. Given its role as a backend integration layer within a broader digital health ecosystem, the impact of SMILHIS cannot be evaluated in isolation. Its effectiveness is inherently linked to the performance and maturity of other system components with which it interfaces, such as EMRs, national/local registries, and other external systems. SMILHIS's benefits would include the establishment of governance structures, adaptation of data standards and protocols, improved architecture, and preliminary demonstrations of data exchange between systems. At this stage, the key performance indicators (KPIs) needed to measure the effectiveness/impact of SMILHIS are limited and would require further refinement as both the technology and the entire ecosystem mature.

### Pilot 1: Pulilan, Bulacan

The pilot implementation of SMILHIS in Pulilan, Bulacan, included a focused evaluation of its impact on the municipal sanitary permit application process. To measure the effectiveness of SMILHIS, a time-and-motion study was conducted to systematically compare the duration and effort required for applicants to complete the permitting process before and after the integration of SMILHIS. This study aims to generate evidence on the potential efficiency improvements brought about by interoperability and process streamlining, particularly in local government workflows that have traditionally relied on paper-based or fragmented systems.

**Figure 7:**
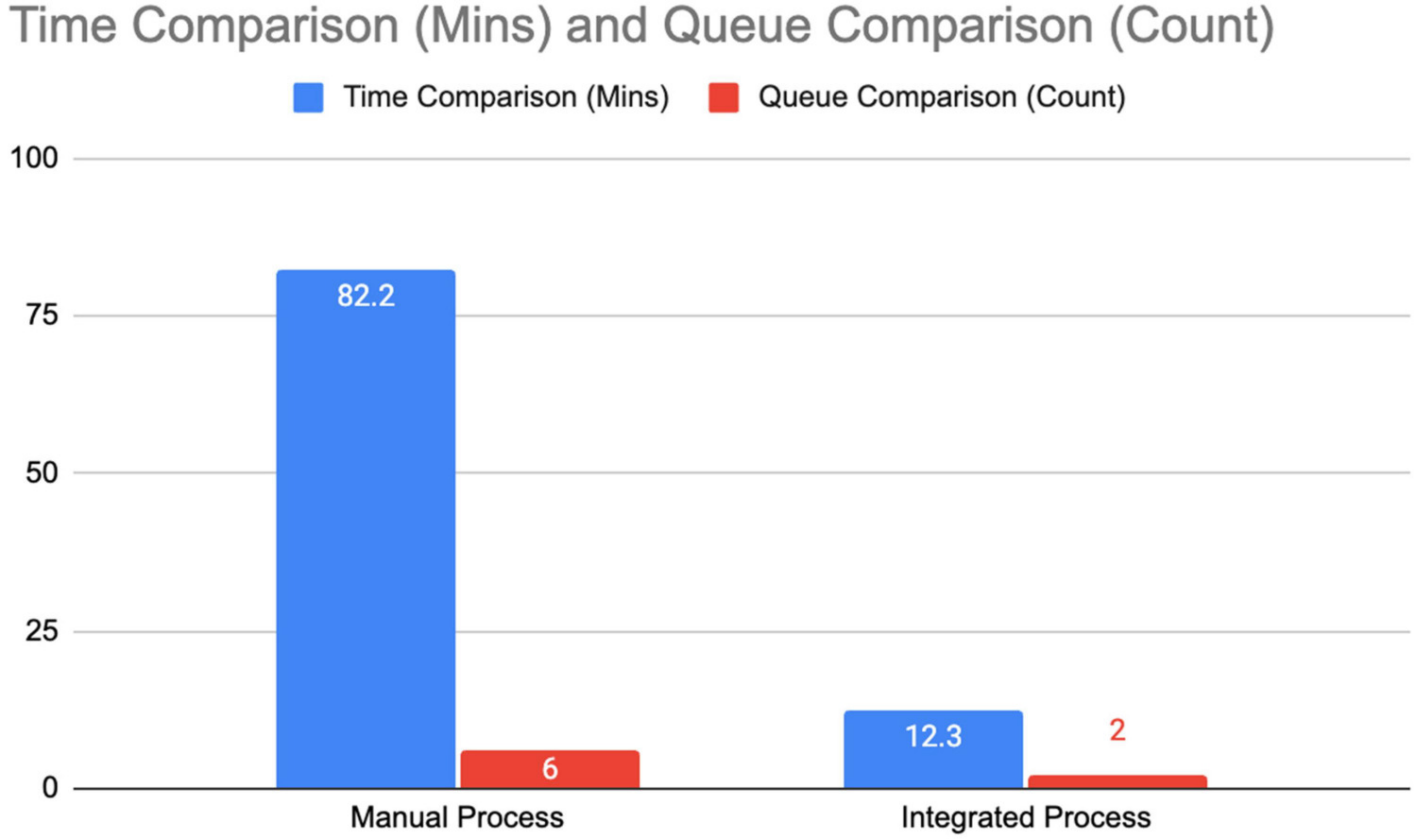
Comparison of manual and integrated process.

Prior to SMILHIS's implementation, the sanitary permit application process involved multiple manual steps across various departments, resulting in long queues, redundant data entry, and limited visibility into the status of applications. Applicants often needed to physically visit several offices, submit duplicate documents, and return multiple times to complete the process. The time and motion study revealed that this legacy process could take several hours to days, depending on staff availability and the completeness of the documents. In contrast, following the deployment of SMILHIS, which enabled real-time data exchange and a unified digital record across relevant offices, the average completion time for applications was significantly reduced. Applicants were able to accomplish the same output in a shorter amount of time, and municipal staff reported fewer administrative bottlenecks. The results of the time and motion study, as shown in Figure 7, demonstrate how the SMILHIS implementation reduced the application period from 82.2 min–12.3 min. This is the result of reducing the number of times an applicant queues (from 6 times–2 times). The reduction in manual steps (manual collection of laboratory results, manual validation of applicants, and manual reading of laboratory results) also contributed to the reduction of application time.

### Pilot 2: Cagayan de Oro

The pilot implementation of SMILHIS in Cagayan de Oro yielded significant insights, particularly in terms of establishing foundational digital health registries, strengthening governance structures, and optimizing system architecture. One of the most notable outcomes was the successful consolidation of key health registries—specifically for patients, facilities, and health workers into standardized, interoperable formats. This enabled a more unified and reliable source of truth across systems, which had previously relied on *ad hoc* and siloed databases. With SMILHIS serving as an integration backbone, these registries are now centrally managed, reducing data duplication and improving accuracy across use cases. The impact of SMILHIS can be seen in the transformation of manual patient registries into digital ones, as well as the presence of localized health facility and health worker registries. In terms of quantitative measures, we have recorded that a total of 650,000 individual patient records and over 23 health facilities have been included in the patient and health facility registry of the pilot SMILHIS implementation in Cagayan de Oro.

**Figure 8:**
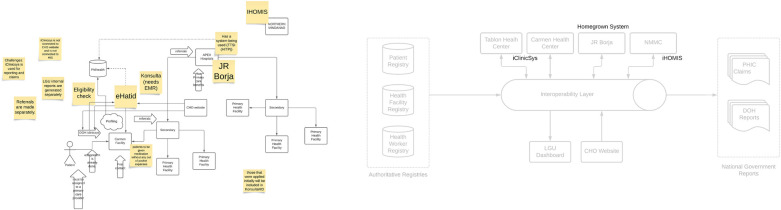
Comparison of architecture (Cagayan de Oro).

Another tangible result was the shift in architectural design. Prior to adopting SMILHIS, each application—whether for electronic medical records, health reporting, or public health programs—required direct point-to-point integrations with multiple other systems. This resulted in a complex web of bespoke interfaces, which increased technical debt and made system upgrades or replacements cumbersome. In contrast, the SMILHIS-enabled architecture follows a hub-and-spoke model, where applications connect to a central layer of interoperability. This significantly reduced the number of required integrations, improved scalability, and enabled faster onboarding of new systems. By decoupling data sources from consuming applications, the new architecture not only simplified maintenance but also laid a more robust foundation for future expansion, including integration with national digital health infrastructures. The change in architecture is more clearly visible in Figure 8.

### Pilot 3: Pangasinan

In Pangasinan, the SMILHIS implementation has led to the successful development and operationalization of a service and bed capacity dashboard, ensuring real-time visibility of the health system and facilitating coordination. This dashboard aggregates data from multiple health facilities within the province, providing up-to-date information on bed availability, referral services, and facility readiness. Prior to this implementation, the referral process was largely manual and dependent on fragmented communication channels, often resulting in inefficient patient transfers. With the dashboard in place, health personnel can now make informed decisions, optimizing resource allocation and improving patient outcomes during routine care and emergencies. Over 72 health facilities, including infirmaries, clinics, and hospitals, were recorded in the health facility registry.

The pilot implementations of SMILHIS have demonstrated the technical and operational feasibility of deploying an LHIE platform across various LGU types and use cases. These deployments validated SMILHIS as a flexible, scalable architecture capable of adapting to varying organizational, infrastructural, and regulatory contexts. Throughout the pilots, several systemic challenges were encountered; however, iterative implementation across different LGUs allowed for the development of targeted mitigation strategies embedded within the SMILHIS framework.

a. **Network Infrastructure Constraints:** The variable quality of internet connectivity, especially in geographically isolated and disadvantaged areas, posed a significant barrier to real-time data exchange. To address this, the SMILHIS team coordinated with the DICT to enable LGUs to utilize national infrastructure resources, including the GovCloud platform. Additionally, data signal heat map surveys were conducted to identify viable third-party internet service providers (ISPs) in proximity to health centers, guiding procurement and network deployment planning.
b. **Standards Conformance and Localization:** SMILHIS implementations adhered to national interoperability standards, including HL7 FHIR and the PHCDI. Technical support was provided to ensure conformance with these standards while allowing for localized adaptations through the profiling of FHIR resources based on specific LGU use case requirements, thereby maintaining semantic consistency while enabling operational flexibility.
c. **Data Governance and Privacy Compliance:** Recognizing the regulatory demands of the Data Privacy Act (DPA) of 2012, each pilot site established a TWG composed of LGU stakeholders and subject matter experts. The TWG was tasked with developing governance structures, including data-sharing agreements, local privacy policies, and standard operating procedures, to guide implementation in compliance with national legal frameworks. These non-technical interventions were integrated with the SMILHIS technical deployment to ensure long-term adoptability, accountability, and sustainability of the LHIE infrastructure.

Overall, the SMILHIS initiative operates not only as a technical solution for health information interoperability but also as a policy-aligned framework that addresses the sociotechnical dimensions of digital health system adoption at the local level.

## Conclusion and recommendations

Implementing SMILHIS across three diverse pilot sites provided critical insights into the challenges and opportunities of deploying interoperable digital health systems in LGUs. One key lesson learned is that the LGU's digital maturity significantly influences the ease of adoption and scaling of health information systems. While some LGUs had pre-existing EMRs and HIS, others relied heavily on paper-based processes, which required more extensive capacity-building and change management interventions. Stakeholder engagement emerged as another crucial factor, as the success of an interoperable health data ecosystem depends not only on technical implementation but also on the willingness of LGU officials, healthcare workers, and private sector partners to embrace digital transformation. Furthermore, the need for standardized data governance policies became evident, particularly in ensuring that data privacy, security, and interoperability frameworks align with national regulations and global best practices.

A significant takeaway from the Pulilan, Bulacan pilot was that streamlining citizen-to-government interactions through interoperability layers can drastically improve service efficiency. Digitalizing regulatory processes, such as the sanitary permit application, demonstrated how system integrations between government offices can reduce processing time, enhance data security, and minimize human intervention in sensitive processes. The Cagayan de Oro implementation underscored the importance of establishing robust HIE mechanisms to enable real-time patient referrals between primary care centers, LGU-operated hospitals, and regional referral facilities. The pilot emphasized the need to develop authoritative registries to support the implementation of UHC. Meanwhile, the Pangasinan pilot highlighted the value of health service visibility in improving patient access to care and optimizing facility utilization. The development of a real-time health referral service dashboard proved instrumental in helping citizens navigate healthcare services while enabling LGUs to identify resource gaps and allocate health system investments more efficiently.

The SMILHIS pilots also revealed several technical and operational challenges that must be addressed to scale the system nationwide. Interoperability gaps between legacy systems remain a pressing issue, mainly as some LGUs rely on proprietary or siloed health IT solutions that are not aligned with nationally mandated data exchange protocols. Additionally, infrastructure constraints, including unstable internet connectivity and a lack of technical capacity in some LGUs, hinder the adoption of digital technologies. The need for sustained technical support and training was evident, as healthcare personnel required continuous education on EMR adoption, health informatics, and cybersecurity best practices. Addressing these issues will require stronger national policy mandates enforcing data standardization and strategic investments in digital infrastructure and workforce capacity-building. Adopting an open architecture is another challenge that LGUs must address to ensure that the HCPNs within the LGU are open to digital technologies.

The non-technical components of SMILHIS require a techno-governance approach to address key gaps in data governance mechanisms and capacity building among LGU employees. Effective data governance ensures that health data management, security, and interoperability policies are upheld across different LGU health information systems, aligning with national policies and regulatory frameworks. By integrating policy-driven data governance frameworks with targeted training programs, SMILHIS fosters organizational readiness, enhances system usability, and promotes a culture of responsible data stewardship, ultimately supporting efficient and ethical digital health transformation at the LGU level.

While this paper provides a detailed account of the design, development, and early implementation of SMILHIS across selected pilot sites, several limitations and caveats must be acknowledged. First, the findings and observations are primarily based on early-stage deployments and may not fully reflect the long-term operational challenges or outcomes that arise once the system scales. Second, the experiences from the LGUs, though diverse, may not be representative of all LGU contexts in the Philippines, particularly those with limited digital infrastructure or governance capacity. Additionally, the availability and quality of data during the pilot phases were dependent on existing systems and local readiness, which may have influenced the scope of integrations and the functionality achieved.

Looking ahead, there is significant potential for expanding SMILHIS beyond the pilot sites to a national scale, particularly by integrating the system with the NHDR. Additionally, expanding the interoperability framework to include private healthcare facilities would strengthen LGU-wide HCPNs and improve patient continuity between public and private health sectors. Another key expansion area is leveraging PhilSys integration further to enhance data integrity and authentication within the SMILHIS ecosystem. Another area that would be considered is the expansion of the registries to include external integration with other registries, such as Philsys or the NHFR. Internally, SMILHIS would benefit from having a more effective KPI measurement mechanism. Future research and evaluation efforts will be necessary to assess the system's impact, sustainability, and adaptability over time and in various settings.

In summary, the SMILHIS project has demonstrated the transformative potential of open, interoperable, and standards-based health information exchanges in strengthening LGU healthcare systems. By addressing system fragmentation, data silos, and inefficient referral mechanisms, the project aligns with the broader goals of UHC in the Philippines. However, sustained collaboration between LGUs, national health agencies, technology vendors, and development partners will be essential to achieve full-scale nationwide adoption. Future initiatives must focus on refining system interoperability, strengthening data governance frameworks, and expanding digital health literacy to ensure that SMILHIS remains a scalable, future-proof solution for local health system digital transformation.

## Data Availability

Publicly available datasets were analyzed in this study. This data can be found here: https://zenodo.org/records/15605748.

## References

[1] Philippines, C. o. Universal Health Care Act (2019).

[2] Asuncion R. Institutionalizing Digital Health to Promote Universal Health Care. Manila: Congressional Policy and Budget Research Department (2022). (Thomas Payne, 2019).

[3] Aragona J. National Health Data Repository Framework. Manila: Philhealth (2022).

[4] Payne T. Status of health information exchange: a comparison of six countries. J Glob Health. (2019) 9(2):020427. 10.7189/jogh.09.020427 31673351 PMC6815656

[5] Aird T Holditch C. An analysis of a novel Canadian pilot health information exchange to improve transitions between hospital and long-term care/skilled nursing facility. J Integr Care. (2022) 30(4):399–412. 10.1108/JICA-03-2022-0022

[6] Lei J Wen D Zhang X Li J Lan H Meng Q Enabling health reform through regional health information exchange: a model study from China. J Healthc Eng. (2017) 2017(1):1053403. 10.1155/2017/1053403 29065565 PMC5387839

[7] Kiourtis A Graziani A Mavrogiorgou A Symvoulidis C Mavrogiorgos K Kyriazis D. A health information exchange protocol supporting bluetooth-based messages. 2021 International Conference on Information Systems and Advanced Technologies (ICISAT), IEEE (2021).

[8] Vidakis K Mavrogiorgou A Kiourtis A Kyriazis D. A comparative study of short-range wireless communication technologies for health information exchange. 2020 International Conference on Electrical, Communication, and Computer Engineering (ICECCE), IEEE (2020).

[9] Symvoulidis C Mavrogiorgou A Kiourtis A Marinos G Kyriazis D. Facilitating the exchange of health information in medical emergencies. 2021 International Conference on e-Health and Bioengineering (EHB), IEEE (2021).

[10] Kiourtis A Mavrogiorgou A Kleftakis S Kyriazis D Torelli F Martino D Categorization of health determinants into an EHR paradigm based on HL7 FHIR. International Conference on Information and Communication Technologies for Ageing Well and e-Health, Springer Nature Switzerland, Cham (2021).

[11] Kouremenou E Kiourtis A Kyriazis D. A Data Modeling Process for Achieving Interoperability. International Conference on e-Health and Bioengineering, Springer Nature Switzerland, Cham (2023).

[12] Mavrogiorgou A Kiourtis A Touloupou M Kapassa E Kyriazis D. Internet of medical things (IoMT): acquiring and transforming data into HL7 FHIR through 5G network slicing. Emerg Sci J. (2019) 3(2):64–77. 10.28991/esj-2019-01170

[13] Craig A. Digital Health and Universal Health Coverage: Opportunities and Policy Considerations for Low- and Middle-Income Pacific Island Countries and Territories. Auckland: Pacific Health (2023).

[14] Oranje M. Exploring the Effects of Digital Technologies in Health Financing for Universal Health Coverage: A Synthesis of Country Experiences and Lessons. Oxford: Oxford Open Digital Health (2024). 10.1093/oodh/oqae016 PMC11932407 40230965

[15] OpenHIE. (2025) Accessed from OpenHIE: Available online at: https://ohie.org/

[16] National Health Facility Registry. (n.d.). Accessed from National Health Facility Registry: Available online at: https://122.53.181.141/VActivefacilitiesList?start=61

[17] HL7 FHIR. (n.d.) Accessed from HL7 FHIR: Available online at: https://hl7.org/fhir/

[18] Zuñiga P Del Mundo J Felizmeno E Mendoza M Zuñiga R. Proof of concept implementation of an enterprise service bus for health information exchanges. Philipp Eng J. (2020) 41(1):27–38.

[19] Batangan, e. a. SMILHIS: Smarter and Integrated Local Health Information System (n.d.). Accessed from ehealth PH: Available online at: https://ehealth.ph/smilhis/

